# Public Health Report of the Secretary of the Treasury

**Published:** 1916-01

**Authors:** 


### Public Health Report of the Secretary of the Treasury.

The annual report of the Secretary of the Treasury as it relates to the Public Health Service contains numerous recommendations bearing on the functions of that organization and evidences the great interest of this department in the extension and expansion of the governmental agencies for the protection of the public health.

In the development of general public health work, according to the Secretary, there is great need of additional medical officers. The number of requests for advice and assistance in health problems received from States and municipalities during the past year has far exceeded that in any similar period in the history of the Service, but the limited number of officers available for the work has prevented in many instances compliance with these requests.

The field investigations, the Secretary states, have served as a stimulus to State and local health agencies, and every effort should therefore be made to encourage and turn to practical account the interest in health matters awakened in the general public. For this reason an increase in the appropriation for field work is requested.

An additional building for the hygienic laboratory is urgently needed. The work of this institution has been greatly extended, particularly as it relates to the examination of viruses, serums and analogous products, a vast market for which has been recently created abroad. The safeguarding of these therapeutic agents requires great accuracy and precision and overcrowding is a serious handicap. In order that the public health may be better protected, an annual appropriation of $15,000 is recommended to be expended in carrying out the provisions of the law relating to the examination of these products.

The United States is the only government of importance which does not provide for the care and isolation of lepers. The establishment of a national leprosarium where the numerous lepers, most of whom are native-born Americans, may be properly segregated and treated, thereby eliminating a menace to the health of others, is urged.

The further recommendations of the Secretary relate to the need of additional clerical assistance in order to meet the demands which are increasingly made on the Public Health Bureau.

